# The Bioactivity of Xylene, Pyridine, and Pyrazole Aza Macrocycles against Three Representative *Leishmania* Species †

**DOI:** 10.3390/pharmaceutics15030992

**Published:** 2023-03-20

**Authors:** Álvaro Martín-Montes, Álvaro Martínez-Camarena, Alberto Lopera, Irene Bonastre-Sabater, M. Paz Clares, Begoña Verdejo, Enrique García-España, Clotilde Marín

**Affiliations:** 1 Departamento de Parasitología, Instituto de Investigación Biosanitaria (IBS. Granada), Hospitales Universitarios de Granada, Universidad de Granada, Severo Ochoa s/n, 18071 Granada, Spain; 2 Instituto de Ciencia Molecular (ICMol), Universidad de Valencia, C/Catedrático José Beltrán 2, 46980 Paterna, Spain

**Keywords:** *Leishmania*, chemotherapy, pyridine, pyrazole, macrocycles, mechanism of action, SOD inhibition

## Abstract

Due to the urgent need for finding effective and free of secondary effect treatments for every clinical form of Leishmaniasis, a series of synthetic xylene, pyridine and, pyrazole azamacrocycles were tested against three *Leishmania* species. A total of 14 compounds were tested against J774.2 macrophage cells which were models for host cells, and against promastigote and amastigote forms of each studied *Leishmania* parasite. Amongst these polyamines, one proved effective against *L. donovani,* another one for *L. braziliensis* and *L. infantum,* and another one was selective solely for *L. infantum.* These compounds showed leishmanicidal activity and reduced parasite infectivity and dividing ability. Action mechanism studies gave a hint that compounds were active against *Leishmania* due to their ability to alter parasite metabolic pathways and reduce (except Py33333) parasitic Fe-SOD activity.

## 1. Introduction

Leishmaniasis is a group of diseases caused by a protozoan parasite that belongs to the genus *Leishmania*. These parasites possess a life cycle composed of two forms. The extracellular one, known as promastigote, serves the parasite as an infective form transmitted by the phlebotomine vector bite; its length ranges from 15 to 20 µm and a flagellum is present. The intracellular form, called amastigote, is located inside infected cells and is in charge of parasitic cell division and dispersion in the vertebrate host. This form is round, smaller (2–5 µm), and lacks flagellum.

There are 21 species that belong to this genus and all of them are pathogenic in humans. Each species causes a certain clinical manifestation in the human host, which can be grouped into three main forms: Cutaneous Leishmaniasis (CL), a form that courses with a single skin alteration or indeed several, such as ulcers that leave a lifelong scar when cured; Mucocutaneous Leishmaniasis (ML), a clinical affection that can cause deformations due to the damage caused to the facial connective tissue, that leaves wounds vulnerable to secondary infections; and Visceral Leishmaniasis (VL), the most life-threatening form that can be fatal if not attended on time and can cause damage to important organs, such as the liver and spleen. These illnesses are endemic in tropical and subtropical areas, where many characteristics of these countries and immunological weakness of the population aggravate the disease impact [1].

**Figure 1:**
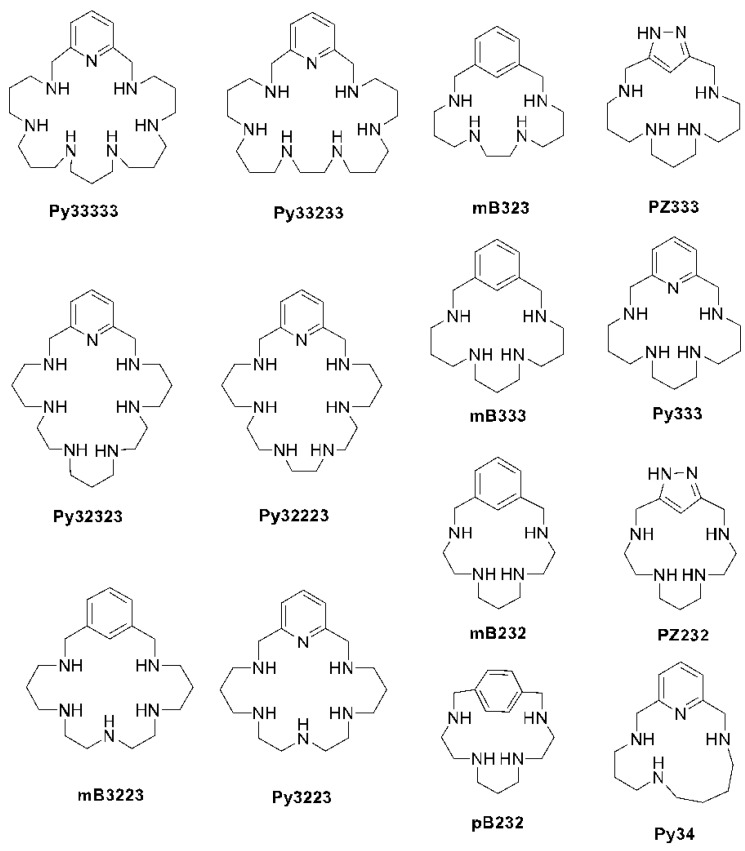
Chemical structure of the tested polyazamacrocyclic compounds.

One of the main reasons for the disease impact is the lack of treatments, as current ones are based on outdated drugs that are effective but toxic and have a long and difficult administration schedule. Therefore, the need to find new compounds to be used as treatments is compelling. These new drugs should be stable at high temperatures, easy to store, affordable, and non-toxic [2]. In this work, we evaluate a group of synthesized macrocycles (Figure 1) as potential leishmanicidal agents.

In this respect, in the last few years we have explored several families of polyamine molecules such as leishmanicidal agents [3,4,5,6,7]. The polyamines explored had different topologies as linear, cyclic, tripodal, etc. Even though the action mechanism may be multitarget, the compounds with the best activity had the common feature to inactivate the superoxide dismutase of the parasites. In view of this point, we have now selected a batch of macrocyclic polyamines of the cyclophane family having different spacers (p-xylene, m-xylene, 2,6-dimethyl pyridine, 3,5-dimethyl-1*H*-pyrazole), numbers of nitrogen atoms, and lengths of the hydrocarbon chains interconnecting the nitrogen donors and we explore their behavior against three Leishmania species, each one as a pathogenic agent for each clinical form: *L. donovani* for visceral leishmaniasis (VL), *L. infantum* for cutaneous leishmaniasis (CL), and *L. braziliensis* for mucocutaneous leishmaniasis (ML) using a variety of assays. Moreover, we study the capability of these compounds to inhibit the SOD of the parasite and we perform molecular dynamic studies to shed light on how the hit compounds may interfere with the enzyme in order to block its antioxidant activity.

## 2. Materials and Methods

### 2.1. Chemistry

All reagents and chemicals were obtained from commercial sources and used as received. Solvents used for the chemical synthesis were of analytical grade and used without undergoing further purification.

### 2.2. Parasite Strain and Culture

Three *Leishmania* species were studied, each one as a pathogenic agent for each clinical form: *L. donovani* for VL, *L. infantum* for CL, and *L. braziliensis* for ML. Their extracellular forms were harvested in Roux Flasks with 75 cm^2^ of surface (Corning, Corning, NY, USA) with MTL (Medium Trypanosomes Liquid) supplemented with 10 % of inactive calf serum and stored at 27 °C (air atmosphere), following the methodology described in Gonzalez et al. 2005 [8].

### 2.3. Cell Culture and Cytotoxicity Studies

Macrophages are cells naturally infected by *Leishmania* parasites. For that reason, they were used as host cell models, specifically the J774.2 macrophage cell line [European Collection of Authenticated Cell Cultures (ECACC) number 91051511]. Cells were used to test the cytotoxicity of the new compound (establishing IC_50_ via testing different drug concentrations on cells) and they were used for parasite infectivity assays [8,9].

### 2.4. In Vitro Activity Assays

When the new compounds showed acceptable levels of cytotoxicity, they were tested against parasite extracellular forms. Assaying compounds on promastigote forms does not yield determinant information, as promastigotes are present in the definitive host for a very short period of time. However, the obtained data from that experiment helps to decide if a drug should progress during the screening process.

In order to perform this experiment, promastigotes were harvested in 24-well microplates and different drug concentrations were added in order to reach 12.5, 25, 50, and 100 µM and maintained at 27 °C for 48 h. They were counted after that time was elapsed in a Neubauer haemocytrometric chamber.

A similar method was used for the amastigote assay. First, J774.2 macrophages were harvested in 24-well microplates with round coverslips at the bottom, where cells will attach. Once cells are fixated, they are infected with promastigotes (10 per each macrophage cell) and compounds were added to reach the same final concentrations as in the previous experiment. After one day, promastigotes transform into amastigotes and microplates are maintained for two days at 37 °C. Once that time is elapsed, coverslips are collected and fixated to a microscope slide and dyed with Giemsa. Amastigotes are then counted using a microscope from a total of 200 random macrophages [8,9].

### 2.5. Infectivity Assays

Following a similar method as the described one in the amastigote experiment, macrophages were harvested and infected with promastigotes in their stationary phase. The test dosage was IC_25_ for this experiment. The coverslips were collected every 2 days for 10 days and counted in order to determine if drugs affected promastigote infection and amastigote division within the cells by measuring the infection rate (percentage of infected cells) and amastigote number per infected cell [10].

### 2.6. SOD Inhibition Assay

Superoxide dismutase (SOD) is a key enzyme for cell survival against oxidative stress. Host cells induce oxidative bursts to create reactive oxygen species (ROS) within parasite cells that are toxic to them. For that reason, *Leishmania* cells rely on SOD to counteract this defense. While vertebrate SOD is linked to a copper, zinc (Cu/Zn-SOD), or manganese atom (Mn-SOD), trypanosomatid SOD is linked to an iron atom (Fe-SOD). This difference makes SOD a perfect target for drugs: if they are able to reduce its activity, cell survival will be compromised. For testing, if drugs are able to reduce this enzymatic activity, promastigotes are largely harvested and then stressed in the same culture medium without calf serum, in order to trigger stress and make them excrete SOD. This SOD is collected and semi-purified by a method described in Martín-Montes et al. 2017 [10]. Excreted Fe-SOD was mixed with the different reagents of the Beyer and Fridovich method [11] and measured in a UV spectrophotometer twice. The second measurement took place after 10 min under direct light and constant stirring. Commercial human SOD (Cu/Zn-SOD) (Sigma^®^, St. Louis, MO, USA) activity was also measured for comparison.

### 2.7. Metabolite Excretion

Active compounds can trigger metabolic alterations within parasite mitochondria. Those alterations can be proven by observing alterations in the excreted metabolites quantity. Trypanosomatids excrete different metabolites to the medium in order to get rid of the carbonate skeleton instead of oxidizing glucose to CO_2_ and water as vertebrates do. Each metabolite is produced in a certain area of the mitochondrion and by a certain enzyme, thus, it can be inferred that these particular zones or enzymes are being affected by drug action [12]. For this experiment, cultures of each species with an initial concentration of 5 × 10^5^ cells/mL were stored at 27 °C for 72 h with IC_25_ as the test dosage. After this time elapsed, cultures were centrifuged at 400× *g* for 10 min and supernatants were collected and examined by ^1^H-NMR.

### 2.8. Rhodamine Assay

One of the typical alterations that compounds can trigger is a depolarization of the mitochondrial membrane. This can be easily measured using rhodamine staining due to the affinity of this colorant to the mitochondrial membrane. Following the same culture method as in the previous experiment, cultures were centrifuged at 800× *g* for 10 min and pellets were collected and stained with Rho 123 (Sigma-Aldrich^®^) and analyzed via flux cytometry. Changes in fluorescence intensity can be interpreted as membrane depolarizations. Those changes were calculated using the formula: VI = (TM − CM)/CM. VI stands for Variation Index, TM is the median fluorescence for treated parasites, and CM is the median fluorescence for untreated parasites [13].

### 2.9. Theoretical Calculations

The computational study of the ligands started with the building of their structures employing the *xleap* software included in AMBER16 (Assisted Model Building with Energy Refinement) software [14]. The protonation degree of the ligands at physiological pH (H_2_**PZ333**^2+^, H_2_**PZ232**^2+^, and H_3_**Py33333**^3+^) and the distribution of the protonated amines along the receptors are deduced by analysis of the speciation studies. The representative structure of the Fe-SOD enzyme was taken from the Protein Data Bank (PDB ID: 2GPC) [15,16]. To build up the L:Fe-SOD complexes, the ligands were approximated to the active center of the enzyme using LEaP software [14]. 

Once the systems were built, they were energetically minimized, and after an equilibration stage at 300 K, a total of 10 ns (10 cycles of 10^6^ steps each) molecular dynamics (MD) was performed. During the modeling, a distance restraint was applied between the heavy atoms of the active site residues and the heavy atoms of the compounds, using the ⟨r^−6^⟩^−1/6^ average of all the interaction distances to atoms of the groups. All the studies were performed using AMBER16 software [14]. The organic compounds were modeled using the *gaff* [17] force field while the *ff14SB* [18] one was used for the Fe-SOD. Finally, a series of 10 minimum energy conformers were selected and then energetically optimized again. The MD simulation trajectory was analyzed using R [19] and the CPPTRAJ module [20] within AmberTools17, while PyMOL [21] was employed for visual inspection and to create molecular graphics.

### 2.10. Synthesis of ***mB323*** and ***mB333***

The new compounds **mB323** and **mB333** were obtained by the reaction of either the pertosylated amine 1,5,8,12-tetrakis(p-tolylsulfonyl)-1,5,8-12-tetrazadodecane (**mB323**) or the pertosylated amine 1,5,9,13-tetrakis(p-tolylsulfonyl)-1,5,9,13-tetrazatridecane (**mB333**) with the m-xylene in a 1:1 molar ratio using potassium carbonate (K_2_CO_3_) as the base in refluxing acetonitrile (CH_3_CN). Detosylation of the macrocycles was carried out with HBr/HAc/PhOH obtaining the final products as hydrobromide salts.

*3,7,10,14 Tetraaza-1(2,6)-benzenacyclopentadecaphane* (**mB323**·4HBr) ^1^H NMR (D_2_O, 300 MHz), δ_H_ (ppm): 7.66 (s, 1H), 7.51 (s, 3H), 4.29 (s, 4H), 3.40 (s, 4H), 3.32 (s, 4H), 3.18–3.02 (m, 4H), 2.05–1.90 (m, 4H). ^13^C NMR (D_2_O, 75.43 MHz), δ_C_ (ppm): 133.50, 132.81, 131.62, 131.50, 44.70, 43.94, 42.33, 22.62. Anal. Calcd. for C_16_H_32_N_4_·4HBr (600.03 g/mol): C, 32.02; H, 5.38; N, 9.34. Found: C, 31.8; H, 5.5; N, 9.5.

*3,7,11,15 Tetraaza-1(2,6)-benzenacyclohexadecaphane* (**mB333**·4HBr) ^1^H NMR (D_2_O, 300 MHz), δ_H_ (ppm): 7.67 (s, 1H), 7.55 (s, 3H), 4.33 (s, 4H), 3.18–3.07 (m, 12H), 2.13–2.00 (m, 6H). ^13^C NMR (D_2_O, 75.43 MHz), δ_C_ (ppm): 132.00, 131.66, 130.82, 130.67, 49.93, 43.04, 42.87, 21.62, 20.66. Anal. Calcd. for C_17_H_34_N_4_·4HBr (614.06 g/mol): C, 33.25; H, 5,58; N, 9.12. Found: C, 33.5; H, 5.6; N, 9.3.

## 3. Results and Discussions

### 3.1. Synthesis of the Compounds

**Scheme 1:**
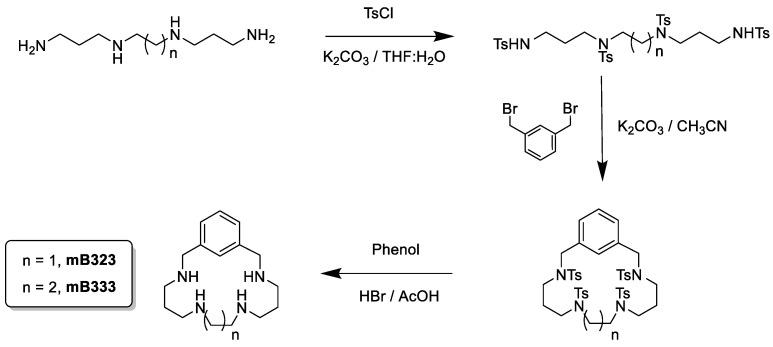
Synthetic scheme for the macrocycles **mB323** (n = 0) and **mB333** (n = 1).

The synthesis of all polyaminic compounds (Figure 1) is based on a modification of the Richman–Atkins procedure [22], which consists in the reaction of the pertosylated amines with the corresponding aromatic spacers in a 1:1 molar ratio using potassium carbonate (K_2_CO_3_) as the base in refluxing acetonitrile (CH_3_CN) (Scheme 1). Detosylation of the macrocycles was carried out with HBr/HAc/PhOH. The final compounds were isolated either as hydrobromide or hydrochloride salts. While the synthesis of the **mB323** and **mB333** macrocycles is presented here for the first time, the synthesis of the other compounds is collected in refs [23,24,25,26,27,28,29,30,31].

### 3.2. In Vitro Activity and Infectivity Assays

An initial roster of 14 macrocyclic compounds was analyzed. In order to establish drug effectiveness, the Nwaka criteria [32] were followed, being an IC_50_ value close to 10 µM and, more importantly, a Selectivity Index (SI) 20 times higher than the one obtained for the reference drug (Glucantime^®^) the parameters to be considered. SI is expressed as the mathematical formula: J774.2 Macrophage IC_50_/Parasite IC_50_. This indicates that to be selected, a drug does not necessarily need to be more lethal to parasites than the reference drug, because lesser cytotoxicity can lead to a higher SI, and therefore it can become a promising initial option.

As shown in Table 1, Table 2 and Table 3, most of the studied compounds did not present a prominent activity, and of those that showed some effect, it was limited to only one parasite, with the exception of **PZ232** which was active against *L. infantum* and *L. braziliensis*. This is remarkable as these two species are somewhat distant in terms of phylogeny: *L. infantum* and *L. donovani* belong to the *L. donovani* species complex, while *L. braziliensis* belongs to the *L. mexicana* complex. One particular factor that aids these compounds and is considered as selective is the extremely low cytotoxicity they show, with some macrophage toxicity IC_50_ values of 1500, 1292, and 984.9 µM, for instance.

Leishmanicidal action was measured in both forms of the parasite life cycle. The most important value is the one obtained for amastigotes as this is the most lasting form within the host. Values for this form obtained with active compounds are close to 200 times the reference drug value. 

**Figure 2:**
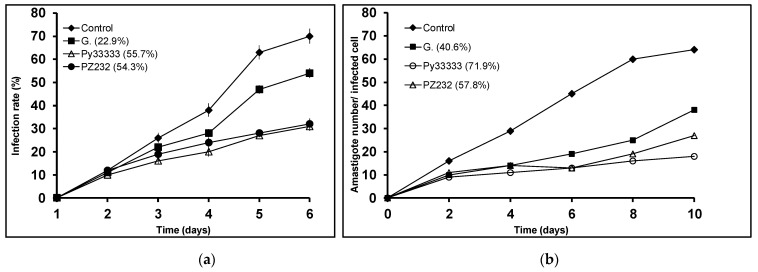
Infection rate charts (**a**) and amastigote count per infected cell charts (**b**) for *L. infantum*.

**Figure 3:**
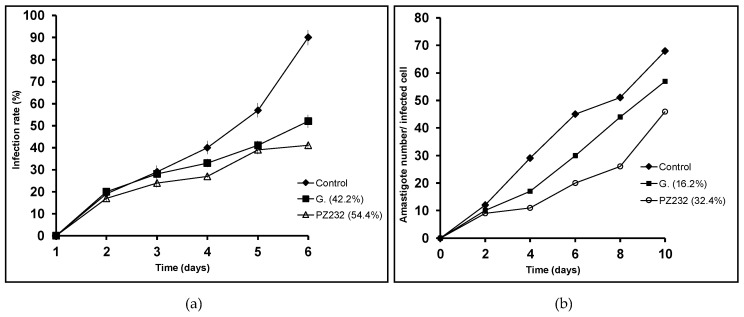
Infection rate charts (**a**) and amastigote count per infected cell charts (**b**) for *L. braziliensis*.

**Figure 4:**
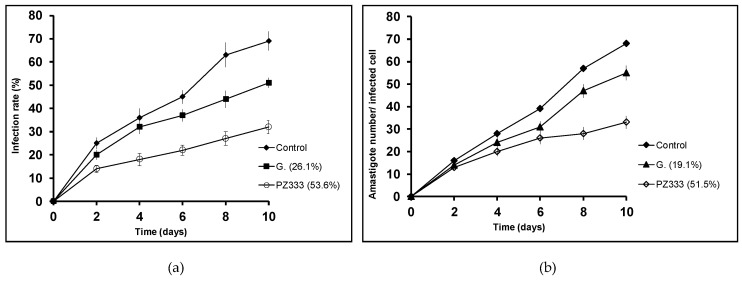
Infection rate charts (**a**) and amastigote count per infected cell charts (**b**) for *L. donovani*.

Since the studied compounds are supposed to reduce the parasite’s ability to infect macrophage cells and to divide within them, infectivity experiments were then carried out. Two features were taken into account: infection rate (percentage of infected cells) and amastigote number in the infected cell. Only the active compounds in the in vitro activity assays were considered for the infectivity experiments, that is, **Py33333** for *L. infantum*, **PZ333** for *L. donovani,* and **PZ232** for *L. infantum* and *L. braziliensis*. As expected, both measured parameters were lower in all the studied compounds than in the control (see Figure 2, Figure 3 and Figure 4).

The assayed compounds were more effective than Glucantime (reference drug) reducing the parasite infection and division abilities. Regarding infection rates at 10 days, every studied drug produces a reduction of over 50%, which is a promising result to take into account. The lowest reduction in amastigote count in infected macrophages was 32.4% for *L. braziliensis* when treated with **PZ232**, while the highest reduction was achieved by **Py33333** against *L. infantum*. The following experiments aimed to get an insight of which particular stage of metabolism is being altered and thus, a preliminary idea of the mechanism of action.

### 3.3. SOD Inhibition Assays

**Figure 5:**
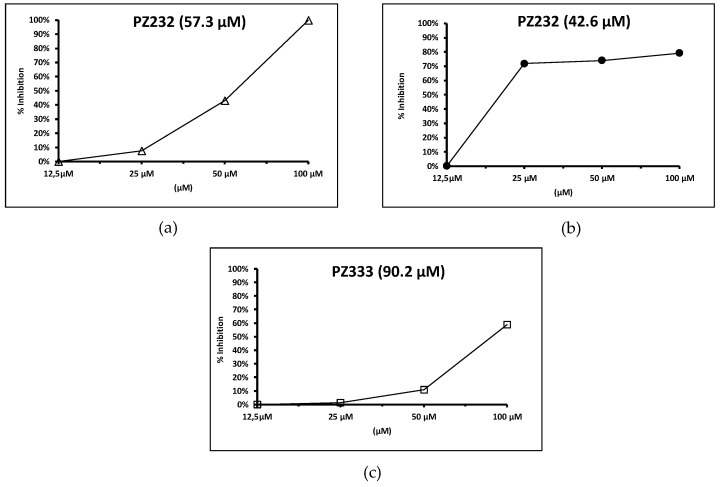
Fe-SOD activity inhibition percentages for *L. infantum* (**a**), *L. braziliensis* (**b**), and *L. donovani* (**c**). Drug IC_50_ for enzymatic activity is displayed in parenthesis. Note: only results for one compound are displayed for *L. infantum*, as the other active one (**Py33333**) showed no inhibition in parasitic Fe-SOD.

Though expected, a reduction of the Fe-SOD activity is not necessarily produced by the compounds that show significant activity in the in vitro activity and infectivity assays. However, this is our case: a remarkable reduction of the Fe-SOD activity of the parasites was observed in the presence of the studied compounds (see Figure 5) while, what is even more interesting, they did not affect human Cu/Zn-SOD activity at all. Nevertheless, one of the active compounds for *L. infantum*, coded as **Py33333**, did not affect parasite enzymatic activity, so its action mechanism seems to be more related to metabolic alterations that will be discussed later on. In this study, all compounds proved to alter normal parasite metabolism. On the other hand, the different parasites were affected to a different extent by a given drug. For example, while *L. braziliensis* Fe-SOD activity is notoriously affected at low concentrations, much higher concentrations are required to yield the same result on *L. infantum*’s Fe-SOD activity.

### 3.4. Metabolite Excretion Assays

**Figure 6:**
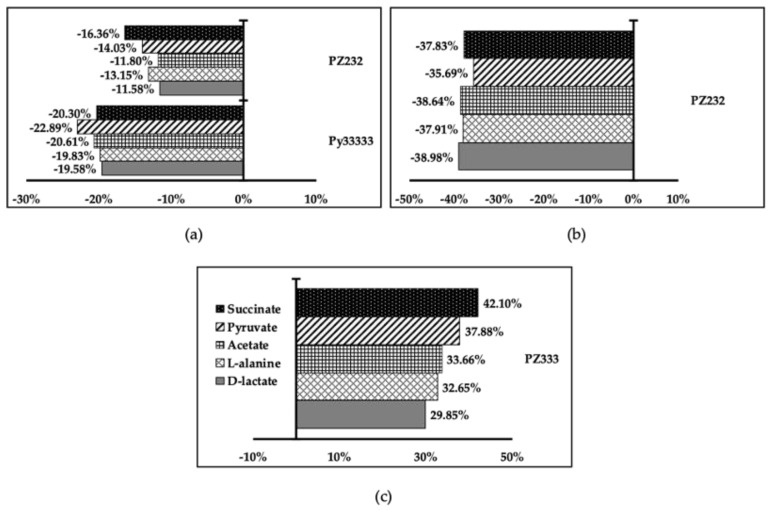
Parasite metabolic production and excretion variation graphs for *L. infantum* (**a**), *L. braziliensis* (**b**), and *L. donovani* (**c**).

All compounds reduced excretion of all metabolites except **PZ333** in *L. donovani*, where it enhanced excretion of all metabolites (see Figure 6), which can be understood as an acceleration of glucose intake from the medium, leading to nutrient exhaustion for the parasite. In the rest of the cases, a global reduction of metabolite production and excretion could mean that glucose intake is not taking place, leading to parasite starvation.

### 3.5. Rhodamine Assay

**Figure 7:**
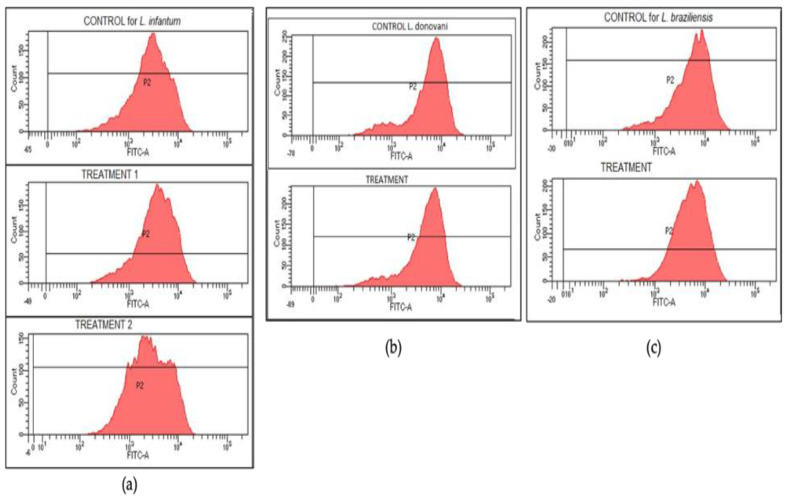
Rhodamine intensity graphs for each studied parasite. No depolarization took place, hence the similarity between the control (upper graphs) and treatment graphs for *L. infantum* (**a**), *L. braziliensis* (**b**), and *L. donovani* (**c**).

A rhodamine assay was conducted in order to find out if drug effectiveness was related to mitochondrial membrane depolarization. Independently of the drug activity, no significant reductions in rhodamine intensities were found. Graphs for control and treatment groups are similar in every case (see Figure 7), which clearly indicates that no depolarization takes place and compounds act in other mechanisms.

Results indicate that selected products can alter the metabolic route of the parasites without depolarizing the mitochondrial membrane and also deprive parasites from their oxidative defenses reducing their Fe-SOD activity without altering host Cu/Zn-SOD, which supports the initial result of the low toxicity that compounds provoke in J774.2 macrophage cells.

### 3.6. Theoretical Calculations

#### 3.6.1. Preliminary Description of the Active Center of the Fe-SOD Enzyme

*T. cruzi* Fe-SOD consists of a dimeric structure, each of which monomers present an independent active center. In this, the metal ion is coordinated by histidines 27, 75, and 164 by aspartic acid 160 and by a molecule of water (or a hydroxide anion, depending on the oxidation state of the Fe) in a trigonal bipyramidal geometry. The substrates’ approach to this active center occurs through a funnel in which histidine 31 and tyrosine 35 serve as the gateway and restrict the access of small molecules toward the catalytic cavity. The proton exchange between the active center and the exterior of the protein is guaranteed by a hydrogen bond network that involves tyrosine 35, glutamine 71, and the coordinating water molecule.

#### 3.6.2. Nature of the Interaction Macrocycle: Fe-SOD

**Figure 8:**
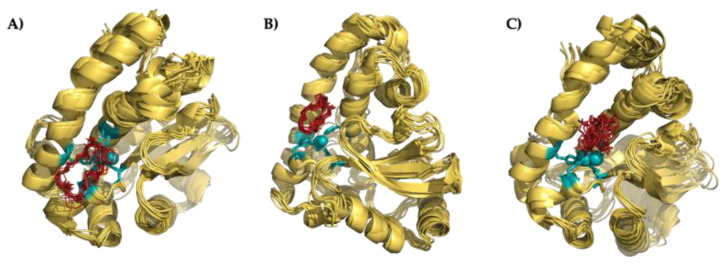
Superimposition of the 10 minimum energy conformers of the interaction between **Py33333** (**A**), **PZ232** (**B**), and **PZ333** (**C**) with Fe-SOD.

Attending to modeling results, the three ligands considered in this study are able to interact with the Fe-SOD enzyme through some of the reactive points of its active center. As shown in Figure 8, the superimposition of the minimum energy conformers shows a very similar structure, in which the macrocycles are directly bonded to different residues of the active center. In all cases, the interaction is based on: (a) ionic bonds between the protonated amino groups of the ligand and anionic residues of the enzyme (for instance, the carboxylic group of glutamic acid 163 in the case of **Py33333**) or (b) hydrogen bonds between the amino groups of the ligands and the heteroatoms of the residues of the Fe-SOD (for example, the non-coordinating nitrogen of the histidine 164 and a nitrogen of histidine 31 in the case of **PZ232**).

#### 3.6.3. Interaction Mode of the Macrocycles

**Figure 9:**
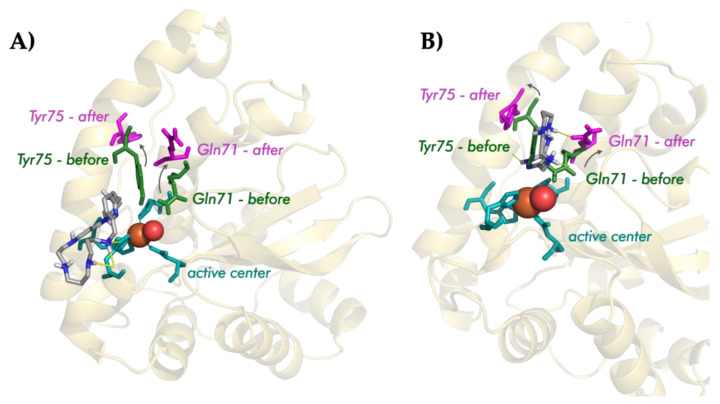
Detail of the interaction between **Py33333** (**A**) and **PZ232** (**B**) with Fe-SOD for the minimum energy conformer.

Though the three macrocycles interact with the FeSOD enzyme, their interaction mode differs depending on the structure of the ligands: while the pyrazole ligands tend to interact with the residues of the hydrogen-bond network, the pyridine one interacts directly with the histidines of the active center. The detail of the interaction can be observed in Figure 9.

The analysis of the interaction of the pyrazole macrocycles with FeSOD shows a similar behavior for both of them. In the minimum energy conformers, both pyrazole ligands are located between the residues glutamine 71 and tyrosine 75, blocking access to the funnel of the active center of the enzyme, and disrupting its hydrogen bond network. Moreover, the ligands tend to interact with histidines 31 and 164 of the active center of the Fe-SOD enzyme using one of its protonated amino groups. 

On the other hand, the pyridine ligand **Py33333** interacts by means of hydrogen bonds and directly with the residues of the active center of the enzyme (mainly by interacting with histidine 164). In this case, the interaction is more direct. The different modes of interaction can be clearly seen in Figure 8, in which the location of the ligand in the active center of the Fe-SOD has been highlighted. 

**Figure 10:**
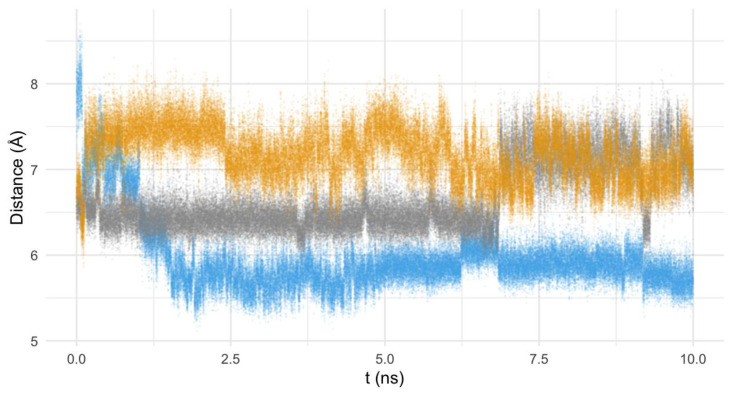
Distance (Å) from the mass center of **Py33333** (in blue), **PZ232** (gray), and **PZ333** (yellow) to the active center of the Fe-SOD in the function of the time of calculation (ns).

**Figure 11:**
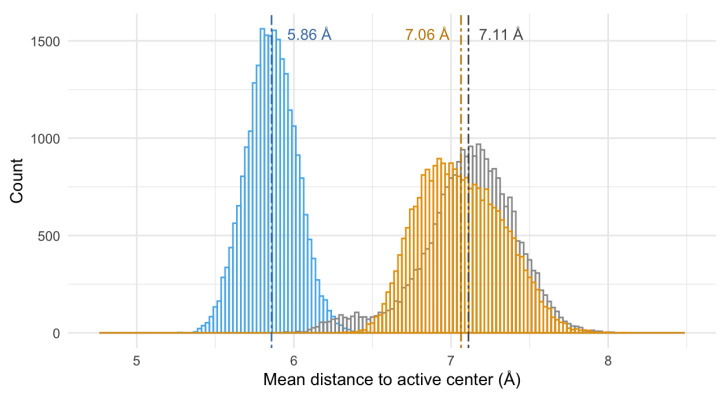
Histogram of the mean distance (Å) from the mass center of **Py33333** (in blue), **PZ232** (gray), and **PZ333** (yellow) to the active center of the Fe-SOD for the last 2.5 ns of calculation. The dashed lines represent the mean value of the histogram of each compound.

The similar interaction mode of both pyrazole ligands and the divergence of the pyridine behavior of the pyridine macrocycle are also consistent in time (and not only in the structure of the minimum energy conformers), as evidenced by the analysis of the mean distance of the molecule to the active center of the enzyme along the theoretical simulation (see Figure 10 and Figure 11). Figure 10 shows the mean distance of the macrocycles to the active center of the enzyme during the 10 ns modeling, while the histogram of the distances to the active center for the last 2.5 ns has been represented in Figure 11.

By paying attention to the mean distance values of the macrocycles to the active center of Fe-SOD along the MD we can see that, though the pyridine system initially starts further away from the active center (7.9(4) Å), it rapidly approaches the proximities of the Fe atom (5.9(1) Å), settling in this location (as can be clearly seen in Figure 10). On the other hand, the pyrazole ligands tend to move away from the active center, though their distance was initially shorter (the **PZ232** ligand moves from 6.6(1) to 7.1(3) Å and the **PZ333** one from 6.7(2) to 7.1(1) Å). The convergence to the same interaction points of the pyrazole ligands with the enzyme can be clearly seen graphically in Figure 8, but also in Figure 10, since distances and trajectories are almost identical for **PZ333** and **PZ232** for the last 2.5 ns of modelling. In this sense, the histograms shown in Figure 11 point to the same behavior of the pyrazole macrocycles during their interaction with Fe-SOD: both **PZ333** and **PZ232** present very similar mean values and relative populations of distances to the Fe-SOD’s active center.

**Figure 12:**
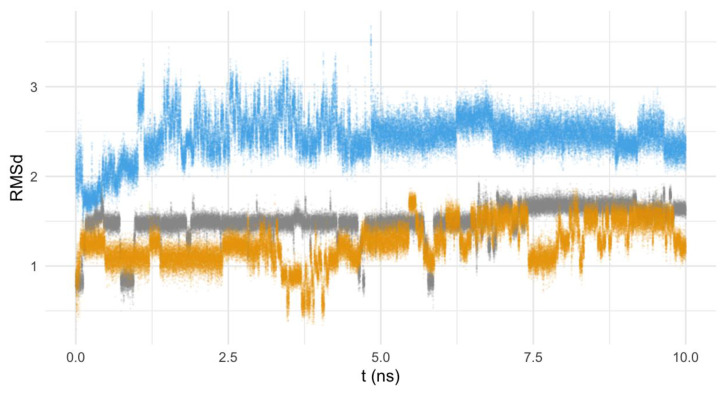
RMSd of **Py33333** (in blue), **PZ232** (gray), and **PZ333** (yellow) in the function of the time of calculation (ns).

Finally, the mobility of the ligands in the interaction position with the FeSOD’s active center seems to be slightly higher for the pyridine ligand, when compared with the pyrazole ones (Figure 12). This can be explained by the larger structure of **Py33333**, and the subsequent higher degree of freedom for this compound.

Two general conclusions can be derived from the MD study:

(a) The three compounds are able to interact with the active center of the enzyme. Indeed, the nature of the interactions is the same in the three cases, i.e., hydrogen and ionic bonds;
(b) The interaction mode differs depending on the structure of the macrocycle: the pyrazole ligands tend to interact with the residues of the hydrogen-bond network, while the pyridine one interacts directly with the histidines of the active center.

## 4. Conclusions

In total, 3 out of the 14 synthetic macrocycles studied have been proven as potential chemotherapy agents against *Leishmania* by inhibiting their antioxidant defense relying on Fe-SOD and altering their metabolism, possibly by augmenting or inhibiting glucose intake from the medium. Two of the compounds had pyrazole as a spacer and the other one pyridine, while none of the macrocycles containing xylene spacers reached the required standards. Three compounds had showed activity against the viscerotropic *Leishmania* spp. studied, presenting a wider activity spectrum than the other active ligands, which were only active to one of them. In the case of ligand **Py33333**, its activity is entirely explained by its capability to inhibit parasitic metabolism leading to starvation, as it was ineffective at inhibiting parasite Fe-SOD. However, its overall anti-parasitic activity is higher than all other products, highlighting that compound anti-parasitic activity is not necessarily linked to the Fe-SOD inhibition ability. Another remarkable trait of the studied compounds is that they were outstandingly selective to Fe-SOD, as they did not affect vertebrate Cu/Zn-SOD, which constitutes a desirable quality as Fe-SOD is one of the most important targets in *Leishmania* chemotherapy. 

## Figures and Tables

**Table 1:** Antiparasitic activity, macrophage toxicity, and selectivity index for *L. infantum*. Results are the means of three separate experiments.

Compound Codes	Compound IC_50_ (μM)		Macrophage Macrophage Toxicity IC_50_ (µM)	Selectivity Index	
	Promastigote Forms	Amastigote Forms		Promastigote Forms	Amastigote Forms
**Glucantime**	50.1 ± 2.3	84.6 ± 4.3	175.8 ± 10.6	3.5	2
**Py3223**	191.9 ± 5.8	nd	165.3 ± 4.9	1 (0)	nd
**Py33233**	484.0 ± 10.7	nd	1292.3 ± 77.5	3 (1)	nd
**Py32323**	90.6 ± 3.8	nd	85.4 ± 4.6	1 (0)	nd
**Py32223**	93.4 ± 3.7	nd	57.7 ± 1.9	1 (0)	nd
**Py33333**	8.51 ± 0.6	1.7 ± 0.2	1500.0 ± 75.3	176 (44)	882 (441)
**mB323**	95.6 ± 4.8	nd	428.5 ± 22.6	4 (1)	nd
**mB333**	86.1 ± 3.7	nd	915.9 ± 51.6	11 (3)	nd
**mB232**	205.1 ± 30.7	nd	667.1 ± 88.5	3 (1)	nd
**pB232**	367.2 ± 10.5	nd	719.1 ± 20.5	2 (1)	nd
**PZ333**	70.0 ± 0.7	nd	984.9 ± 72.8	14 (4)	nd
**PZ232**	13.0 ± 0.4	4.9 ± 0.4	673.8 ± 66.3	52 (13)	138 (69)
**mB3223**	63.4 ± 2.4	nd	1450.7 ± 89.7	23 (6)	nd
**Py333**	30.4 ± 1.4	nd	309.9 ± 43.8	10 (3)	nd
**Py34**	56.7 ± 2.6	nd	568.9 ± 45.8	10 (3)	nd

**Table 2:** Antiparasitic activity, macrophage toxicity, and selectivity index for *L. braziliensis*. Results are the means of three separate experiments.

Compound Codes	Compound IC_50_ (μM)		Macrophage Toxicity IC_50_ (µM)	Selectivity Index	
	Promastigote Forms	Amastigote Forms		Promastigote Forms	Amastigote Forms
**Glucantime**	51.4 ± 2.5	103.3	175.8 ± 10.6	3	1.7
**Py3223**	290.7 ± 3.8	nd	165.3 ± 4.9	1 (0)	nd
**Py33233**	507.1 ± 8.3	nd	1292.3 ± 77.5	3 (1)	nd
**Py32323**	1634.3 ± 76.4	nd	85.4 ± 4.6	0 (0)	nd
**Py32223**	441.7 ± 5.8	nd	57.7 ± 1.9	0 (0)	nd
**Py33333**	36.5 ± 1.1	nd	1500.0 ± 75.3	41 (14)	nd
**mB323**	2.3 ± 0.5	17.1 ± 1.1	428.5 ± 22.6	186 (62)	25 (13)
**mB333**	209.6 ± 5.3	nd	915.9 ± 51.6	4 (1)	nd
**mB232**	91.7 ± 3.7	nd	667.1 ± 88.5	7 (2)	nd
**pB232**	27.2 ± 0.9	nd	719.1 ± 20.5	26 (9)	nd
**PZ333**	15.7 ± 0.5	nd	984.9 ± 72.8	63 (21)	nd
**PZ232**	5.8 ± 0.2	1.6 ± 0.6	673.8 ± 66.3	116 (39)	421 (211)
**mB3223**	15.6 ± 0.7	nd	1450.7 ± 89.7	93 (31)	nd
**Py333**	26.2 ± 1.1	nd	309.9 ± 43.8	12 (4)	nd
**Py34**	29.2 ± 0.8	nd	568.9 ± 45.8	19 (6)	nd

**Table 3:** Antiparasitic activity, macrophage toxicity and selectivity index for *L. donovani*. Results are the means of three separated experiments.

Compound Codes	Compound IC_50_ (μM)		Macrophage Toxicity IC_50_ (µM)	Selectivity Index	
	Promastigote Forms	Amastigote Forms		Promastigote Forms	Amastigote Forms
**Glucantime**	23.7 ± 0.8	76.4 ± 4.1	175.8 ± 10.6	7	2
**Py3223**	52.5 ± 2.1	nd	165.3 ± 4.9	3 (0)	nd
**Py33233**	583.0 ± 6.9	nd	1292.3 ± 77.5	2 (0)	nd
**Py32323**	130.2 ± 9.5	nd	85.4 ± 4.6	1 (0)	nd
**Py32223**	1000.0 ± 78.4	nd	57.7 ± 1.9	0 (0)	nd
**Py33333**	70.5 ± 5.2	nd	1500.0 ± 75.3	21 (3)	nd
**mB323**	158.1 ± 7.3	nd	428.5 ± 22.6	3 (0)	nd
**mB333**	105.6 ± 7.5	nd	915.9 ± 51.6	9 (1)	nd
**mB232**	171.2 ± 10.4	nd	667.1 ± 88.5	4 (1)	nd
**pB232**	742.9 ± 23.6	nd	719.1 ± 20.5	1 (0)	nd
**PZ333**	1.4 ± 0.3	1.7 ± 0.5	984.9 ± 72.8	704 (101)	579 (290)
**PZ232**	25.0 ± 0.8	nd	673.8 ± 66.3	27 (4)	nd
**mB3223**	126.5 ± 3.8	nd	1450.7 ± 89.7	11 (2)	nd
**Py333**	0.2 ± 0.0	16.8 ± 0.5	309.9 ± 43.8	1549 (221)	18 (9)
**Py34**	28.4 ± 0.6	nd	568.9 ± 45.8	20 (3)	nd

## Data Availability

Data available on request to the authors.

## References

[1] Burza S., Croft S.L., Boelaert M. (2018). Leishmaniasis. Lancet.

[2] Leishmaniasis. https://www.who.int/es/news-room/fact-sheets/detail/leishmaniasis.

[3] Navarro P., Sánchez-Moreno M., Marín C., García-España E., Ramirez-Macías I., Olmo F., Rosales M.J., Gómez-Contreras F., Yunta M.J.R., Gutiérrez-Sánchez R. (2014). In vitro leishmanicidal activity of pyrazole-containing polyamine macrocycles which inhibit the Fe-SOD enzyme of Leishmania infantum and Leishmania braziliensis species. Parasitology.

[4] Marín C., Inclán M., Ramírez-Macías I., Albelda M.T., Cañas R., Clares M.P., Gonzalez-Garcia J., Rosales M.J., Urbanova K., García-España E. (2016). In vitro antileishmanial activity of aza-scorpiand macrocycles. Inhibition of the antioxidant enzyme iron superoxide dismutase. RSC Adv..

[5] Martín-Montes A., Clares M.P., Martín-Escolano R., Delgado-Pinar E., Marín C., Verdejo B., Martínez-Camarena A., Molina-Carreño D., García-España E., Sánchez-Moreno M. (2021). Heterocyclic Diamines with Leishmanicidal Activity. ACS Infect. Dis..

[6] García M.P., García-España E., Blasco S., Soriano C., González-GarcÍa J., Verdejo B., Inclán M. (2014). Compuestos Macrocíclicos de Tipo Escorpiando y su uso Como Antiparasitarios. España Patent.

[7] Arán V., Navarro P., Sánchez-Moreno M., Marín C., Olmo F., Ramirez-Macías I., García-España E., Albelda M.T. (2016). Use of Ester Derivatives of Pyrazole Proton-Ionizable Compounds and the Corresponding Salts Thereof for the Treatment of Chagas Disease and Leishmaniasis. WO 2016/038238, PCT/Es/2015/070658.

[8] González P., Marín C., Rodríguez-González I., Hitos A.B., Rosales M.J., Reina M., Díaz J.G., González-Coloma A., Sánchez-Moreno M. (2005). In vitro activity of C20-diterpenoid alkaloid derivatives in promastigotes and intracellular amastigotes of Leishmania infantum. Int. J. Antimicrob. Agents.

[9] Kirkinezos I.G., Moraes C.T. (2001). Reactive oxygen species and mitochondrial diseases. Semin. Cell Dev. Biol..

[10] Martin-Montes A., Santivañez-Veliz M., Moreno-Viguri E., Martín-Escolano R., Jiménez-Montes C., Lopez-Gonzalez C., Marín C., Sanmartín C., Gutiérrez Sánchez R., Sánchez-Moreno M. (2017). In vitro antileishmanial activity and iron superoxide dismutase inhibition of arylamine Mannich base derivatives. Parasitology.

[11] Beyer W.F., Fridovich I. (1987). Assaying for superoxide dismutase activity: Some large consequences of minor changes in conditions. Anal. Biochem..

[12] Fernández-Becerra C., Sánchez-Moreno M., Osuna A., Opperdoes F.R. (1997). Comparative aspects of energy metabolism in plant trypanosomatids. J. Eukaryot. Microbiol..

[13] Sandes J.M., Fontes A., Regis-da-Silva C.G., de Castro M.C.A.B., Lima-Junior C.G., Silva F.P.L., Vasconcellos M.L.A.A., Figueiredo R.C.B.Q. (2014). Trypanosoma cruzi cell death induced by the Morita-Baylis-Hillman adduct 3-hydroxy-2-methylene-3-(4-nitrophenylpropanenitrile). PLoS ONE.

[14] Case D.A., Betz R.M., Cerutti D.S., III T.E.C., Darden T.A., Duke R.E., Giese T.J., Gohlke H., Goetz A.W., Homeyer N. (2016). AMBER 2016.

[15] Bachega J.F.R., Navarro M.V.A.S., Bleicher L., Bortoleto-Bugs R.K., Dive D., Hoffmann P., Viscogliosi E., Garratt R.C. (2009). Systematic structural studies of iron superoxide dismutases from human parasites and a statistical coupling analysis of metal binding specificity. Proteins Struct. Funct. Bioinform..

[16] Berman H.M., Battistuz T., Bhat T.N., Bluhm W.F., Bourne P.E., Burkhardt K., Feng Z., Gilliland G.L., Iype L., Jain S. (2002). The Protein Data Bank. Acta Crystallogr. Sect. D Biol. Crystallogr..

[17] Wang J.M., Wolf R.M., Caldwell J.W., Kollman P.A., Case D.A. (2004). Development and Testing of a General Amber Force Field. J. Comput. Chem..

[18] Maier J.A., Martinez C., Kasavajhala K., Wickstrom L., Hauser K.E., Simmerling C. (2015). Ff14SB: Improving the Accuracy of Protein Side Chain and Backbone Parameters from Ff99SB. J. Chem. Theory Comput..

[19] R Core Team (2021). R: A Language and Environment for Statistical Computing.

[20] Roe D.R., Cheatham III T.E. (2013). PTRAJ and CPPTRAJ: Software for Processing and Analysis of Molecular Synamics Trajectory Data. J. Chem. Theory. Comput..

[21] Schrodinger LLC (2015). The PyMOL Molecular Graphics System.

[22] Richman J.E., Atkins T.J., Oetle W.F. (1998). Organic Synthesis.

[23] Díaz P., Basallote M.G., Máñez M.A., García-España E., Gil L., Latorre J., Soriano C., Verdejo B., Luis S.V. (2003). Thermodynamic and kinetic studies on the Cu^2+^ coordination chemistry of a novel binucleating pyridinophane ligand. Dalton Trans..

[24] Aguilar J., Basallote M.G., Gil L., Hernández J.C., Máñez M.A., García-España E., Soriano C., Verdejo B. (2004). Stability and kinetics of the acid-promoted decomposition of Cu(II) complexes with hexaazacyclophanes: Kinetic studies as a probe to detect changes in the coordination mode of the macrocycles. Dalton Trans..

[25] Basallote M.G., Doménech A., Ferrer A., García-España E., Llinares J.M., Mañez M.A., Soriano C., Verdejo B. (2006). Synthesis and Cu(II) coordination of two new hexaamines containing alternated propylenic and ethylenic chains: Kinetic studies on pH-driven metal ion slippage movements. Inorg. Chim. Acta.

[26] Belda R., Blasco S., Verdejo B., Jiménez H.R., Doménech-Carbó A., Soriano C., Latorre J., Terencio C., García-España E. (2013). Homo- and heterobinuclear Cu^2+^ and Zn^2+^ complexes of abiotic cyclic hexaazapyridinocyclophanes as SOD mimics. Dalton Trans..

[27] Verdejo B., Basallote M.G., Ferrer A., Mañez M.A., Hernández J.C., Chadim M., Hodacová J., Llinares J.M., Soriano C., García-España E. (2008). Equilibrium and kinetic properties of Cu(II) cyclophane complexes: The effect of changes in the macrocyclic cavity caused by changes in the substitution at the aromatic ring. Eur. J. Inorg. Chem..

[28] Algarra A.G., Basallote M.G., Belda R., Blasco S., Castillo C.E., Llinares J.M., García-España E., Gil L., Máñez M.A., Soriano C. (2009). Synthesis, protonation and Cu(II) complexes of two novel isomeric pentaazacyclophane ligands: Potentiometric, DFT, kinetic and AMP recognition studies. Eur. J. Inorg. Chem..

[29] Belda R., Pitarch-Jarque J., Soriano C., Llinares J.M., Blasco S., Ferrando-Soria J., García-España E. (2013). Intermolecular binding modes in a novel [1+1] condensation ^1^H-pyrazole azamacrocycle: A solution and solid state study with evidence for CO_2_ fixation. Inorg. Chem..

[30] Lopera A., Gil-Martínez A., Pitarch-Jarque J., Verdejo B., Blasco S., Clares M.P., Jiménez H.R., García-España E. (2020). Influence of the chain length and metal: Ligand ratio on the self-organization processes of Cu^2+^ complexes of [1+1] 1H-pyrazole azamacrocycles. Dalton Trans..

[31] Belda R., García-España E., Morris G.A., Steed J.W., Aguilar J.A. (2017). Guanosine-5′-monophosphate polyamine hybrid hydrogels: Enhanced gel strength probed by z-spectroscopy. Chem. Eur. J..

[32] Nwaka S., Ramirez B., Brun R., Maes L., Douglas F., Ridley R. (2009). Advancing drug innovation for neglected diseases—Criteria for lead progression. PLoS Neglect. Trop. Dis..

